# Oral Presentations - Abstracts of the 3^rd^ Brazilian Congress of PRONUCLEO and Regional Meeting Brazil Red Latinoamericana de Reproducción Asistida (REDLARA)

**DOI:** 10.5935/1518-0557.20240046

**Published:** 2024

**P-01. Can the dual trigger protocol impact embryo ploidy?**

**M.L. Montenegro^1^, L.T.S. Rodrigues^2^, C.S. Souza^1^, S.M. Jau^1^, R. Tomioka^3^, M.M. Piccolomini^3^, B.R.L. Moura^2^, E.G.Lo Turco^2^, F.P. Ferreira^1^**

*^1^Neo Vita Clinic, São Paulo, Brazil*

*^2^Urology Department, Human Reproduction Section, São Paulo Federal University, São Paulo, Brazil*

*^3^Lab for Life, São Paulo, Brazil*

**Objective:** To evaluate the impact of the induction of final follicular maturation by the dual trigger protocol on the euploidy of *in vitro* cultured embryos.

**Methods:** In a retrospective cohort study, data were collected from 1102 patients for 36 months who were splitted into 5 groups according to the trigger protocol. The groups were Choriomon group with 18 patients (CG), Gonapeptyl group with 452 patients (GG), Lupron group with 67 patients (LG), Ovidrel group with 87 patients (OG), Gonapeptyl plus Choriomon group with 478 patients (GCG). All patients included in this study underwent the same protocol of controlled ovarian stimulation, in all cycles genetic analysis of the embryos were performed. For statistical analysis of the data, the ANOVA test was applied with a post hoc bon ferroni test. The value of p<0.05 was adopted to differentiate the groups.

**Results:** Regarding total follicle recovery, the CG, OG, GCG groups showed a lower total oocyte recovery compared to the GG and LG groups (7.44±5.29 *vs*. 10.75±5.30 *vs*. 10.58±5.28 *vs.* 13.96±9.26 *vs*. 14.23±7.31, *p*<0.05). The Gonapeptyl group had a higher percentage of oocytes collected compared to the LG, GCG, OG, CG groups, respectively (15±11.85 *vs*. 14.31±12.06 *vs*. 10.77±6.313 *vs*. 10.07±5.88 *vs*. 6.72±6.05, *p*<0.05). The GG group had a higher number of total blastocysts when compared to the GCG and OG groups (5.23±4.49 *vs*. 3.83±2.70 *vs*. 3.14±2.03, *p*<0.05). The GG group had a higher rate of euploid embryos when compared to the GCG group (0.39±0.35 *vs*. 0.31±0.35, *p*<0.05), respectively. In view of these results, we were able to see a positive impact on success rates in a simple protocol such as Gonapeptyl, which presented better results compared to the dual trigger protocol.

**Conclusion:** This study demonstrated that the dual trigger protocol was not superior to other trigger protocols and that when compared to the trigger protocol with Gonapeptyl, the dual trigger had a lower rate of embryonic euploidy.

**P-02. Clinical results comparing time lapse vs other incubators**

**R. Azambuja^1^, F. Wingert^1^, M.R. Hentschke^1^, V.C. Dornelles^1^, V.D. Trindade^1^, N. Vasconcelos^1^, L. Okada^1^, A. Petracco^1^, M. Badalotti^1^**

*^1^Fertilitat - Reproductive Medicine Center, Porto Alegre, Brazil*

**Objective:** The objective of the present study was to compare laboratory results and clinical outcomes between time-lapse and 5% or 20% O2 incubators.

**Methods:** Retrospective observational study performed at a reproductive medicine center, in Brazil, using data collected between 2020 and 2023. A total of 706 fresh IVF cycles with embryo transfer were included. Women aged >42 years old cycles were excluded from analysis. In order to analyze clinical outcomes, only homologous oocytes' cycles were considered. All transfers were performed on day 5 after insemination, at blastocyst stage. The sample was divided into two groups according to the embryo's incubators: Group 1 - Time-lapse (Embryoscope) (n=371) vs. Group 2 - 5% or 20% O_2_ incubators (n=335). Fertilization and blastulation rates, as well as the clinical outcomes were compared between groups. T-Test, U-Mann Whitney and Chi-square tests were performed, considering *p*<0.05.

**Results:** Results are shown in Table 1.

**Conclusion:** There were no differences regarding fertilization, blastulation, implantation, pregnancy and miscarriage rates; however, there was a higher mean of blastocysts per cycle in the Embryoscope group. This could affect the cumulative pregnancy rate between groups, which was not evaluated in this study. More studies including bigger samples as well as cumulative pregnancy rates analysis are needed for further conclusions.

**Table 1:** Comparison between groups analyzed.

	Group ES (n=371)	Group TRIGAS + FORMA (n=335)	*p*
Male Age, mean ± SD	38.6 ± 5.5	38.4 ± 6.2	0.668
Female age, mean ± SD	35.8 ± 3.7	35.5 ± 3.6	0.354
Iseminated eggs, n	2764	2178	-----
Fertilization rate, n(%)	2146 (77.6)	1690 (77.6)	0.994
Blastulation rate, n(%)	1221 (56.9)	940 (55.2)	0.429
Mean blastocyst/cycle, mean ± SD	3.3 ± 2.1	2.8 ± 2.2	0.002
Implantation rate, n(%)	153/513 (29.8)	142/560 (25.4)	0.101
Pregnancy rate, n(%)	153 (46.2)	144 (41.6)	0.216
Clinical pregnancy rate, n(%)	137 (41.4)	127 (36.7)	0.208
Live birth ± Ongoing pregnancy rate, n(%)	112 (33.8)	101 (29.2)	0.186
Miscarriage rate, n(%)	25 (18.3)	22 (17.3)	0.752

**P-03. Impact of embryo storage time after vitrification on pregnancy and implantation rates**

**R.M.S. Costa^1^, R.L. Bossi^2^, M.M. Carneiro^2,3^, Marcos Sampaio^2^**

^1^Faculdade de Medicina Unifenas.

^2^Clínica Origen.

^3^Faculdade de Medicina UFMG.

**Objective:** Embryo cryopreservation became routine in IVF treatments since 1983 optimizing pregnancy and live birth rates. Vitrification has proven to be more effective than slow freezing since provide better survival rates and clinical outcomes. Statistics of freeze/thaw cycles by ESHRE, ASRM and REDLARA demonstrated a continuously increase over the years. Despite vitrification great results many studies failed to elucidate an association between embryo viability, implantation rates and the duration of cryostorage. The aim of this study was to evaluate whether storage time of vitrification had any effects on pregnancy as well as implantation rates.

**Methods:** This retrospective multicentric study included 1568 autologous frozen embryo-transfer (FET) cycles from January 2015 to December 2019. Patients were assigned into 4 groups according to the storage time: 1.0-90 days, 2.91-120 days, 3.121-360 days, and 4.>360 days to evaluate the impact of embryo storage time on pregnancy and implantation rates. Stratification analyses was based on maternal age (less than 35 years, 36-40 years, more than 41 years); stage of cryopreservation (cleavage or blastocyst) and stage of embryo transfer (cleavage or blastocyst).Sample size calculation revealed 412 women would be necessary in the pregnancy and 412 not-pregnant groups conducted to test the difference in embryo storage period between cases with and without clinical pregnancy. Univariate logistic regression was used to assess the association between storage period and pregnancy rates while multiple logistic regression identified variables related to pregnancy rates. Multiple linear regression was used to assess the association between cryostorage time of vitrified embryos, patients age and transfer outcomes. Moreover, further analyses were performed according to variables with Chi-Square test and Wilcoxon Mann-Whiney test (*p*<0.05).

**Results:** Storage duration was inversely associated with pregnancy and implantation rates and were related to patient age and embryo stage at transfer. Duration of cryostorage was categorized in four groups: 0-90 days (n=840, 53%); 91-120 days (n=308, 20%); 121-360 days (n=172, 11%); >360 days (n=248, 16%). Embryos frozen for 91 to 180 days (n=304) demonstrated lower rates of clinical pregnancy rates compared to those frozen for less than 90 days (n=844) (OR 0.668, 95% CI 0.500; 0.888, *p*=0.006). Patient's age at the time of freezing was a crucial factor for pregnancy and implantation rates. Patients aged 36 to 40 (n=573) and those over 41 years old (n=238) had lower rates of clinical pregnancy compared to those under 35 years old (n=757) (OR 0.563, 95% CI 0.438; 0.721, *p*<0.001 for those aged 36 to 40 years and OR 0.319, 95% CI 0.218; 0.462, *p*<0.001 for women over 41 years old). Additionally, women over 40 also had a lower implantation rate when compared to women under 35 years old (OR 0.157, 95% CI 0.037; 0.45, *p*=0.003). Stage of embryo vitrification was also an important factor, indicating that thawed embryos transferred at the cleavage stage (n=1022) had lower rates of clinical pregnancy (OR 0.381, 95% CI 0.298; 0.485, *p*<0.001) and implantation (OR 0.154, 95% CI 0.100; 0.233, *p*<0.001) compared to embryos vitrified, thawed and transferred at the blastocyst stage (n=546).

**Conclusion:** Storage duration, patient age and embryo stage at vitrification may compromise clinical pregnancy and implantation rates after FET. Thus patients should be advised that long storage time after vitrification in cleavage stage embryos in women older than 36 years are associated with lower clinical pregnancy and implantation rates.

stage at vitrification may compromise clinical pregnancy and implantation rates after FET. Thus patients should be advised that long storage time after vitrification in cleavage stage embryos in women older than 36 years are associated with lower clinical pregnancy and implantation rates.

